# Supplementary material for: Spindle Chirp and other Sleep Oscillatory Features in Young Children with Autism
Source: bioRxiv. 2023 Oct 21:2023.06.15.545095. Originally published 2023 Jun 15. Preprint. [Version 2] doi: 10.1101/2023.06.15.545095 (PMC10312722; doi:10.1101/2023.06.15.545095)
Supplement: 1 [file NIHPP2023.06.15.545095v2-supplement-1.pdf]

bioRxiv preprint doi: https://doi.org/10.1101/2023.06.15.545095; this version posted October 21, 2023. The copyright holder for this preprint (which was not certified by peer review) is the author/funder. This article is a US Government work. It is not subject to copyright under 17 USC 105 and is also made available for use under a CC0 license.

Supplementary Figures

Supplementary figure 1- Differences in chirp in children with developmental delay

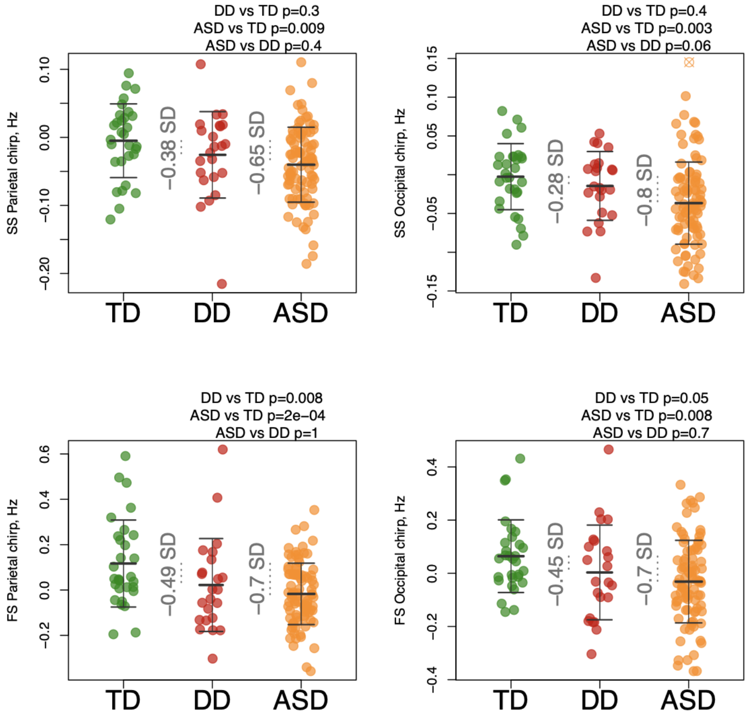

FS = fast spindle; SS = slow spindle

bioRxiv preprint doi: https://doi.org/10.1101/2023.06.15.545095; this version posted October 21, 2023. The copyright holder for this preprint (which was not certified by peer review) is the author/funder. This article is a US Government work. It is not subject to copyright under 17 USC 105 and is also made available for use under a CC0 license.

Supplementary figure 2- Differences in SO and coupling using absolute amplitude threshold for SO detection

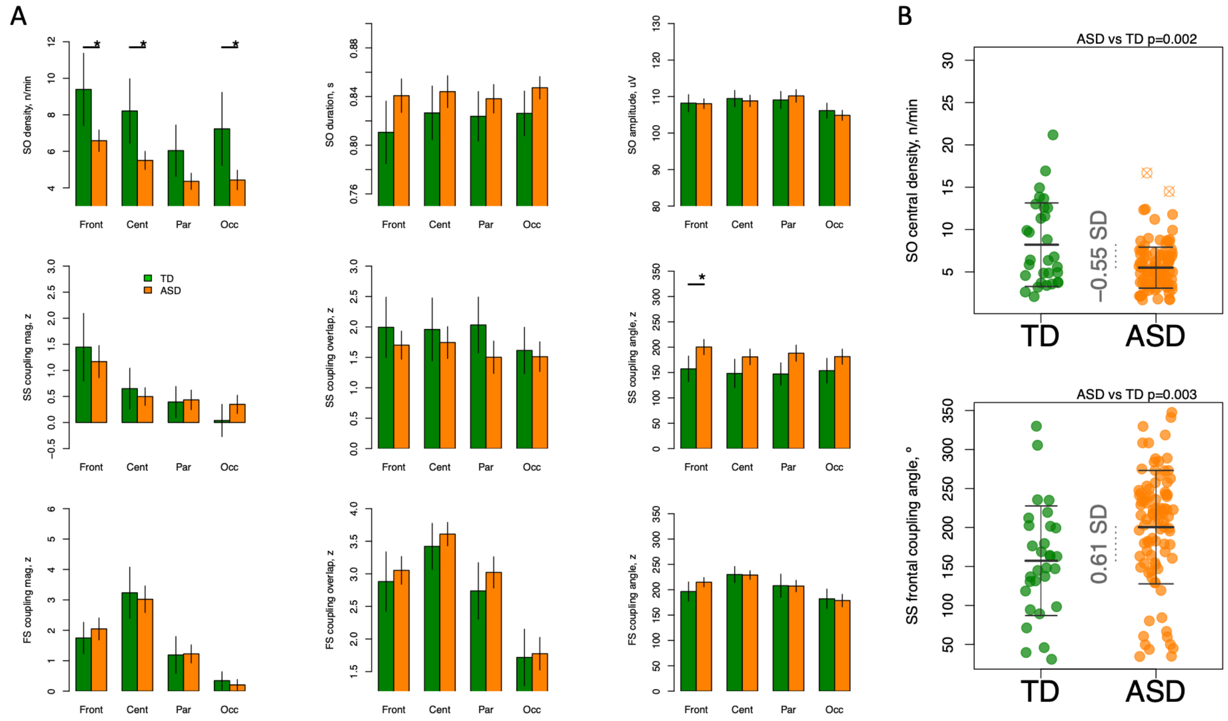

*FS = fast spindle; SO = slow oscillation; SS = slow spindle; * indicates p-value < 0.05*

Supplementary figure 3- Differences in SO and coupling in children with developmental delay using relative amplitude threshold for SO detection

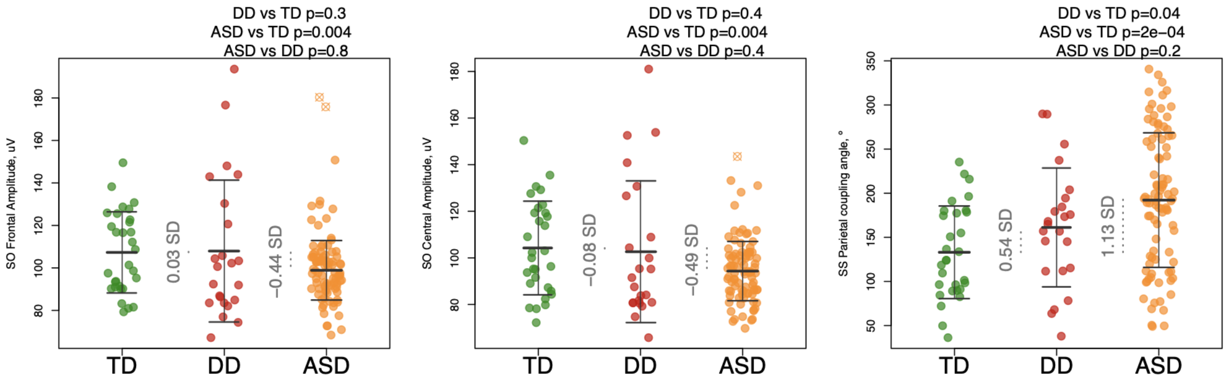

SO = slow oscillation

bioRxiv preprint doi: https://doi.org/10.1101/2023.06.15.545095; this version posted October 21, 2023. The copyright holder for this preprint (which was not certified by peer review) is the author/funder. This article is a US Government work. It is not subject to copyright under 17 USC 105 and is also made available for use under a CC0 license.

Supplementary figure 4- SO phase angle - spindle frequency coupling

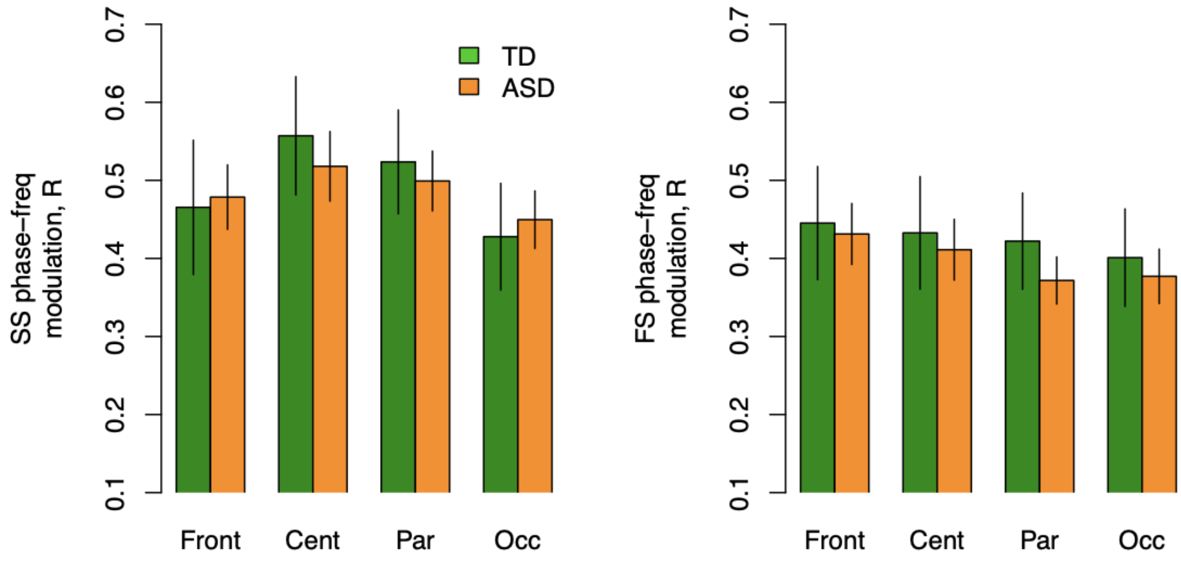

FS = fast spindle; SO = slow oscillation; SS = slow spindle
